# Overwork

**Published:** 1874-12

**Authors:** 


OVERWORK.—The common opinion that excessive mental occupation gravitates toward insanity is not only not verified by facts, but, on the contrary, one of the foremost of living physicians doubts whether alienation of mind is ever the result of overstrain ; it is to physical, not to mental derangement, he thinks, that excessive work of the brain generally gives rise. Insanity, he points out, finds the most suitable material for its development among the cloddish, uneducated classes, while the worst forms of physical diseases are originated and intensified by the educated, overstraining brain-workers.

